# Modulation of the host Th1 immune response in pigeon protozoal encephalitis caused by *Sarcocystis calchasi*

**DOI:** 10.1186/1297-9716-44-10

**Published:** 2013-02-11

**Authors:** Philipp Olias, Anne Meyer, Robert Klopfleisch, Michael Lierz, Bernd Kaspers, Achim D Gruber

**Affiliations:** 1 Institute of Veterinary Pathology, Freie Universität Berlin, Robert-von-Ostertag-Str. 15, Berlin 14163, Germany; 2 Clinic for Birds, Reptiles, Amphibians and Fish, Faculty of Veterinary Medicine, Justus-Liebig-Universität Gießen, Frankfurter Straße 91-93, Gießen, 35392, Germany; 3 Department of Veterinary Science, University of Munich, Veterinärstr. 13, München, 80539, Germany

## Abstract

Pigeon protozoal encephalitis (PPE) is an emerging central-nervous disease of domestic pigeons (*Columba livia* f. *domestica*) reported in Germany and the United States. It is caused by the apicomplexan parasite *Sarcocystis calchasi* which is transmitted by *Accipter* hawks. In contrast to other members of the Apicomplexa such as *Toxoplasma* and *Plasmodium*, the knowledge about the pathophysiology and host manipulation of *Sarcocystis* is scarce and almost nothing is known about PPE. Here we show by mRNA expression profiling a significant down-modulation of the interleukin (IL)-12/IL-18/interferon (IFN)-γ axis in the brains of experimentally infected pigeons during the schizogonic phase of disease. Concomitantly, no cellular immune response was observed in histopathology while immunohistochemistry and nested PCR detected *S. calchasi*. In contrast, in the late central-nervous phase, IFN-γ and tumor necrosis factor (TNF) α-related cytokines were significantly up-modulated, which correlated with a prominent MHC-II protein expression in areas of mononuclear cell infiltration and necrosis. The mononuclear cell fraction was mainly composed of T-lymphocytes, fewer macrophages and B-lymphocytes. Surprisingly, the severity and composition of the immune cell response appears unrelated to the infectious dose, although the severity and onset of the central nervous signs clearly was dose-dependent. We identified no or only very few tissue cysts by immunohistochemistry in pigeons with severe encephalitis of which one pigeon repeatedly remained negative by PCR despite severe lesions. Taken together, these observations may suggest an immune evasion strategy of *S. calchasi* during the early phase and a delayed-type hypersensitivity reaction as cause of the extensive cerebral lesions during the late neurological phase of disease.

## Introduction

*Sarcocystis calchasi* is an apicomplexan parasite and the causative agent of pigeon protozoal encephalitis (PPE), an emerging neurological disease of the domestic pigeon (*Columba livia* f. *domestica*) [1,2]. The definitive hosts of *S. calchasi* are *Accipiter* hawks of which the European subspecies of the Northern goshawk (*Accipiter g. gentilis*) has been experimentally identified to shed large quantities of infectious sporocysts [3,4]. So far the domestic pigeon is the only identified intermediate host of the parasite. Pigeons show a biphasic disease with polyuria, diarrhea and apathy during the schizogonic first phase and severe central nervous signs such as torticollis and opisthotonus associated with severe brain lesions about eight weeks post infection. At the same time mature tissue cysts were present in skeletal muscles. The severity and onset of central nervous signs clearly were dose dependent. In contrast, the intensities of histopathologic lesions and immune cell infiltration in the brains appear to be independent of the amount of administered sporocysts (10^2^-10^5^ per oral dose) [5]. Moreover, no intralesional parasitic stages were observed in H&E histopathology. It therefore appears that the immune system of the pigeon is incapable of preventing infection and an immunopathological basis of the central-nervous lesions has been hypothesized [3]. In this context it can be speculated that *S. calchasi* may benefit from the induction of central-nervous malfunctioning and immobilization as they may influence the rate of parasite’s transmission to its definitive host similar as it is proposed for related apicomplexan parasites [6].

Several avian *Sarcocystis* spp. have been reported to induce central nervous signs (see [1] for overview). Encephalitis is most often reported to be associated with the schizont stage of the parasite’s development. One notable example is *Sarcocystis neurona*. This parasite which is closely related to *S. calchasi* and most probably of avian origin is capable of inducing a central nervous disease in a broad range of avian and mammalian species such as horses, cats, and dogs [7-10]. In many cases and even in extensive lesions the number of intralesional *S. neurona* merozoites and schizonts can be very low. It has been proposed that an immune response triggered by cytokines and metabolites of the parasite may cause the extensive lesions [11]. Recently the presence of *S. neurona* tissue cysts together with schizonts and merozoites has been confirmed for the first time in southern sea otters (*Enhydra lutris nereis*) with encephalitis [12]. More significantly, ovine *Sarcocystis* spp. such as *Sarcocystis tenella* has been found capable of inducing a widespread encephalomyelitis associated with degenerating tissue cysts and prominent central nervous signs [13,14].

Until now the biology of the hosts’ immune response against *Sarcocystis* spp. in general has only scarcely been addressed and whether this genus of parasites may manipulate the immune response similarly to other Apicomplexa is unknown. However, in vitro results suggest that *S. neurona* might be capable of down-modulating the IFN-γ signaling pathway [15,16]. It has therefore been proposed that *Sarcocystis* spp. may use similar evasion strategies than *Toxoplasma gondii*, a well-studied apicomplexan parasite that interferes with the IFN-γ signaling pathway [17,18]. Here, we aimed at investigating the immune response and pathophysiology of PPE due to *S. calchasi* during the schizogonic and late chronic phase of disease associated with central-nervous signs. In most cases we confirmed the presence of parasitic stages in the brains of the pigeons by immunohistochemistry and nested PCR. The cytokine expression profile together with the morphological results of this study may suggest an immune evasion strategy of the parasite that interferes with the Th1 response in the first phase of the disease, while an overstimulated T-cell mediated immune response appears to be characteristic for the second phase of the disease.

## Material and methods

### Samples of pigeons

The samples used for the present study originate from an experimental infection study of *S. calchasi* in domestic pigeons [3]. All experiments were performed under governmental approval (No. Reg 0111/08). The pigeons were orally inoculated with a range of 10^2^ to 3 × 10^6^ sporocysts shed by an experimentally infected Northern goshawk (*A. g. gentilis*) as described previously [5]. The pigeons depicted a biphasic disease. Two animal groups were established for the purpose of this study. The eight pigeons of group A inoculated with 8 × 10^4^ to 3 × 10^6^ sporocysts deceased 7–12 dpi during the schizogonic early phase of disease. The five pigeons of group B inoculated with 10^2^ to 10^4^ sporocysts were euthanized 51–65 dpi in the central nervous late phase of disease. Two additional pigeons (L60, L74) were inoculated with 10^3^ sporocysts, euthanized in the second phase at day 53 and 59 dpi and integrated into group B (Table 1). Five uninfected pigeons were used as reference animals. The brains of all pigeons were removed immediately after death [5]. One half was snap frozen and stored at −80°C until further use. The other half was fixed in 4% neutral-buffered formaldehyde and embedded in paraffin 24 h later. In addition, tissue samples from lung, heart, liver, spleen, kidneys, gizzard and skeletal muscles were taken and processed equally.

**Table 1:** Numbers of immunohistochemically detected schizonts and tissue cysts in five consecutive transversal section of brains of pigeons during first (group A) and second, central nervous phase of disease (group B)

**Group**	**Pigeon ID no.**	**Dose of administered sporocysts**	**Post mortem (dpi)**	**Occurrence of central nervous signs (dpi)**	**Encephalitis**	**No. of**	
						**schizonts**	**tissue cysts**
A	2	3 × 10^6^	7	/	/	1	0
	3	3 × 10^6^	7	/	/	1	0
	4	3 × 10^5^	9	/	/	0	0
	5	3 × 10^5^	9	/	/	0	0
	6	10^5^	8	/	/	0	0
	7	10^5^	12	/	/	3	0
	8	8 × 10^4^	9	/	/	1	0
	9	8 × 10^4^	10	/	/	0	0
B	10	10^4^	51	51	+++	0	8
	11	10^4^	53	52	+++	0	2
	12	10^3^	58	57	+++	0	0
	14	10^2^	65	64	+++	0	0
	15	10^2^	58	57	+++	0	0
	L60	10^3^	53	52	+++	0	4
	L74	10^3^	59	58	+++	0	0

+++, severe.

For details on histopathology of further organs see [5].

### Antibodies against *S. calchasi*

*S. calchasi* sporocysts derived from a Northern goshawk euthanized 14 days after oral infection were used for generation of *S. calchasi*-specific polyclonal antibodies [3]. Washed sporocysts were pretreated with 5% sodium hypochlorite for 30 min, washed and resuspended in 15 mL Roswell Park Memorial Institute medium (RMPI) medium supplemented with 10% fetal bovine serum (FBS) and 15% bovine bile and were incubated for 1 h at 37°C. Vero cells grown in RMPI medium were inoculated with 10^8^ excysted sporozoites and supplemented with FBS, 10 000 IU/mL penicillin and 10 000 μg/mL dihydrostreptomycin. Merozoites were harvested after 12 days by rinsing the monolayer with 10 mL 4°C Hank’s buffered salt solution, washed and resuspended in 2.5 mL PBS and passed through a PD-10 desalting column (GE Healthcare, Freiburg, Germany) as previously described [19]. Purified merozoites were incubated in 4% formalin for 30 min and washed three times in PBS. Standard immunization of two rabbits was conducted with 1 × 10^7^ merozoites each. Histological sections of skeletal muscle infested with tissue cysts and livers infested with merozoites and schizonts of *S. calchasi* from experimentally infected domestic pigeons were used to assess the specificity of the serum.

### Histopathology and immunohistochemistry

Formalin-fixed paraffin-embedded tissue was sectioned at 4 μm, mounted on glass slides and stained with haematoxylin and eosin (H&E).

Immunohistochemistry was used to analyze the prevalence of parasitic stages of *S. calchasi* and expression of MHC-II, CD3 for T-cells and Pax-5 for B-cells in pigeon brains. Serial sections of frozen brain samples were cut at 4 μm, mounted on adhesive glass slides and were fixed in acetone for 10 min and dried for 20 min. Avidin-biotin blocking of the cryostat sections was performed according to the manufacturer’s protocol (Dako North America, Inc., Carpinteria, CA, USA). The slides were washed in PBS containing 0.05% Triton X-100 and blocked with PBS containing 2% BSA and 20% normal goat serum for 30 min. Finally the sections were incubated with mouse-anti-chicken MHC-II specific antibody 2G11 (1:50) for 1 h. The antibody 2G11 has been shown to cross-react with MHC-II of multiple avian and non-avian species [20]. A goat anti-mouse IgG (1:200, Vector Laboratories, Burlingame, CA, USA) was used as secondary antibody. MHC-II immunoreaction was visualized by incubating in ABC solution, followed by HistoGreen-staining (Linaris, Wertheim-Bettingen, Germany) for 4 min at room temperature.

For detection of CD3 and Pax-5, sections of formalin-fixed paraffin-embedded tissue samples were cut at about 2 μm and mounted on glass slides. Consecutive sections were dewaxed in xylene, followed by rehydration in descending graded ethanol. Endogenous peroxidase was blocked by incubating the slides with 0.5% H_2_O_2_ in methanol for 30 min at room temperature. Antigen retrieval was performed using 15 min microwave heating (600 W) in 10 mM citric acid, pH 6.0, containing 0.05% Triton X-100. A polyclonal rabbit antibody specific for the highly conserved ε-chain of human CD3 (1:3000, DAKO, Glostrup, Denmark) and a monoclonal mouse anti-human Pax-5 specific antibody (clone 24, 1:1000, BD Biosciences, San Jose, CA, USA) were diluted in Tris-buffered saline (TBS, 50 mM, pH 7.6) and incubated at 4°C overnight after a blocking step with 50% goat serum in TBS (30 min at room temperature). Both antibodies have been tested on spleen cell populations for cross-reactivity with pigeon T- and B-cells, respectively. Goat anti-rabbit IgG (1:200) and anti-mouse IgG (1:200, Vector Laboratories), respectively, was used as secondary antibody. Color was developed by incubating the slides in avidin-biotin-peroxidase complex (ABC) solution (Vectastain Elite ABC Kit, Vector Laboratories, Burlingame, CA, USA), followed by exposure to diaminobenzidine tetrahydrochloride (DAB, Merck, Darmstadt, Germany). All slides were counterstained with Mayer’s haematoxylin. In each run, negative controls were incubated with irrelevant commercial mouse or rabbit immunoglobulins (BioGenex, Fremont, CA, USA) instead of primary antibodies listed above. No unspecific labelling was detected in any tissue examined.

### RNA isolation, cDNA preparation and DNA isolation

About 40 mg cerebrum of each pigeon was minced into small pieces. RNA was extracted and purified using the NucleoSpin RNA II kit (Macherey-Nagel, Düren, Germany). Concentrations were measured by OD 260 and OD 280 (NanoDrop 1000 Spectrophotometer, Thermo Fisher Scientific, Wilmington, DE, USA). RNA integrity numbers (RIN) above 8.0 were measured in all samples using an Agilent 2100 Bioanalyzer (RNA 6000 Nano Kit, Agilent, Santa Clara, CA, USA) and regarded as good quality [21]. For cDNA synthesis, 50 ng RNA was reverse transcribed using the iScript kit (Bio-Rad, Hercules, CA, USA). DNA was extracted using the NucleoSpin Tissue kit (Macherey-Nagel).

### Nested and quantitative real time (RT) PCR

*S. calchasi* DNA was detected by nested PCR targeting the ITS1 region as described previously [4].

For candidate reference genes, three primer pairs for beta-actin, beta glucuronidase (GUSB) and hydroxymethylbilane synthetase (HMBS) were used (A. Meyer, unpublished observations). In addition, two new primer pairs for ribosomal protein L13 (RPL13) and transferrin receptor protein (TFRC) were designed using Netprimer software (PREMIER Biosoft, Palo Alto, CA, USA) based on a comparative sequence analysis of published mRNA sequences of the domestic chicken (*G. gallus* f. *dom.*) and the zebra finch (*Taeniopygia guttata*) using MEGA4 (Table 2) [22]. In the same way a novel set of primers was designed for 10 cytokines of the domestic pigeon including interleukin (IL) 1, IL-6, IL-7, IL-12, IL-15, IL-18, interferon gamma (IFN-γ), transforming growth factor beta 2 (TGF-β2), LPS-induced TNF-α factor (LITAF), TNF-like ligand 1A (TL1A) and the chemokine IL-8 (Tab. 2). For RT-PCR, the 15 μL reaction mix included 10 μL Brilliant SYBR Green QPCR Master Mix (Applied Biosystems, Carlsbad, CA) with 300 nM of each primer and 5 μL sample cDNA. Cycling conditions were 10 min at 95°C followed by 40 cycles at 30 s at 95°C, 1 min at 58°C and 30 s at 72°C. Reference genes were evaluated to quantify relative cytokine mRNA expression levels. RT-PCR and data were analyzed using the MX 3000P Quantitative PCR System and MX Pro software (Agilent). Each sample was analyzed in triplicate. Nuclease-free water was used as negative controls in each run. Initially, primer efficiencies were determined and primers with an efficiency below 90.0 excluded from further analysis (Table 1). Specificity of amplicons was evaluated against chicken sequences derived from GenBank using MEGA5 (Table 1) and by melting curve analysis. Relative cytokine mRNA expression levels were normalized by the efficiency-corrected ΔΔCt method against the expression of the three most stable reference genes determined by the GeNorm algorithm [23-25]. Data are presented as fold change (FC) in cytokine expression levels in pigeons of group A and B, respectively, normalized to the reference genes and relative to the group of reference animals. Cut off values were set at > 2.0 for increased and < 0.5 for reduced gene expression.

**Table 2:** **Sequences of primers used for RT-qPCR and uncorrected (*p*) sequence distance of obtained pigeon amplicons to chicken mRNA**

**RNA target**	5^′^-3^′ ^primer sequences	**Amplicon size (bp)**	**Primer efficiency (%)**	*p*-distance*	Accession number**
beta-actin	F: AAGGACCTGTACGCCAACAC	211	91.8	0.024	NM_205518
	R: CCTGCTTGCTGATCCACATC				
GUSB	F: GGGGCAAACTCCTTCCG	223	92.2	0.127	NM_001039316
	R: ATCCACCAGCTTGATGTCACTAAC				
HMBS	F: CTGGCCCGGATTCAGAC	154	96.3	0.166	XM_417846
	R: GCTCTTTGGTGAAGAGGCTC				
RPL13	F: CCACAAGGACTGGCAGCG	135	92.0	0.095	NM_204999
	R: ACGATGGGCCGGATGG				
TFRC	F: GCCCTGAATGACAGGATGATG	206	87.3	0.17	NM_205256
	R: GTCCACGTCGCTAGGGCC				
IL-1	F: CGAGAGCAGCTACGCCG	271	99.6	0.176	DQ393270
	R: GCCGCTCAGCACACACG				
IL-6	F: CTGCCCAAGGTGACGGAG	178	97.9	0.3	HM179640
	R: CCAGGTGCTTTGTGCTGTAGC				
IL-7	F: CAGAGTATCGTGACAGATGCTGC	174	101.3	0.111	NM_001037833
	R: ATGAGACTAATGCTGCTTTCCTTC				
IL-8	F: CAAGACGTGAAGCTGACACAGAG	161	99.5	0.117	DQ393275
	R: GGTGCATCAGAATTGAGTTGAG				
IL-12	F: AGTGAAGGAGTTCCCAGATGC	188	90.0	0.194	DQ202328
	R: TTCCAGAGTAGTTCTTTGCCTCAC				
IL-15	F: GAATGCCAGGAACCTGTAATG	246	102.5	0.142	HQ005358
	R: GCATTCCCTCTGTATAACCTTTAC				
IL-18	F: GCCAGTTGCTTGTGGTTCG	160	100.8	0.141	AY775782
	R: TCTACCTGGACGCTGAATGC				
IFN-γ	F: CAGATGTAGCTGACGGTGGAC	276	93.2	0.178	DQ479967
	R: GCTCATGCACAGCTTTGCG				
TGF-β2	F: GAAGAAGCGTGCTCTAGATGC	105	100.1	0.019	ENSGALT00000015664
	R: CATTTCCAGCCAAGATCCC				
LITAF	F: CCCATCTGCACCACCTTCAT	157	100.8	0.252	NM_204267
	R: TGCTGCACATACACAGTCTGAAC				
TL1A	F: CCTGAGTTATTCCAGCAACGCA	285	95.3	0.111	NM_001024578
	R: ATCCACCAGCTTGATGTCACTAAC				

*compared to chicken by MEGA5 software. **of chicken.

### Statistical analysis

Gene expression data were analyzed with the Mann–Whitney-U test using SPSS software, version 20.0 (SPSS, X). Results were considered statistically significant at *p* < 0.05 between two groups of animals.

## Results

### Histopathology and immunohistochemistry

**Figure 1:**
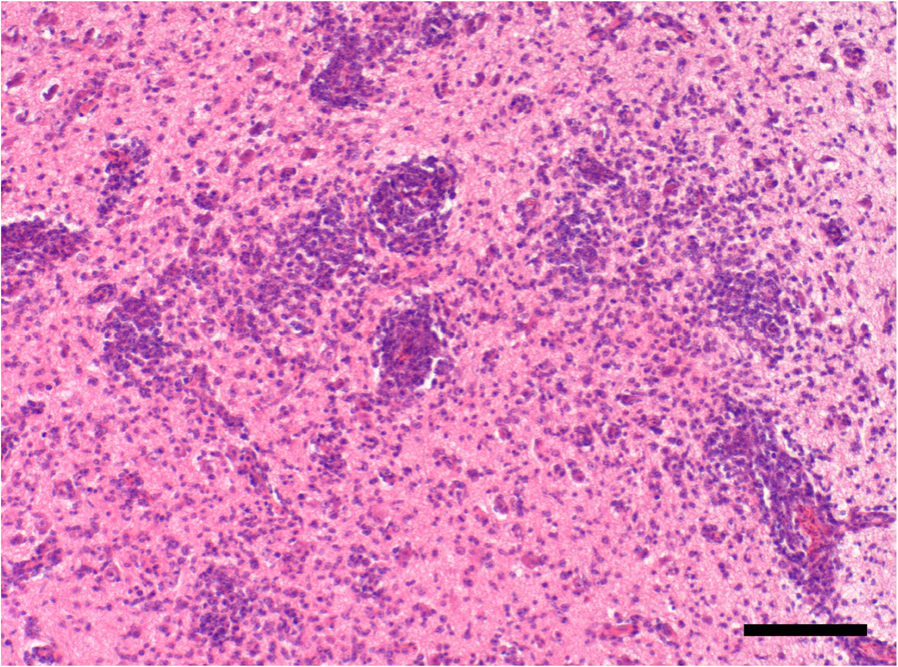
**Encephalitis of pigeon no. 14 infected with 10**^**2 **^**sporocysts and negative for *S. calchasi *by immunohistochemistry and PCR. **Note the extensive mononuclear cell infiltration and perivascular cuffing in the cerebrum. H&E stain. Bar, 100 μm.

Pigeons of group A that died during the schizogonic first phase of disease had no histopathological lesions in the brain. No inflammatory cell response was discernible in any area. Pigeons of group B were euthanized in the neurological second phase of disease. All pigeons showing central nervous signs uniformly had a severe multifocal lymphohistocytic and necrotizing encephalitis with prominent perivascular cuffing (Figure 1) [5]. No pathological lesions were found in the reference animals.

**Figure 2:**
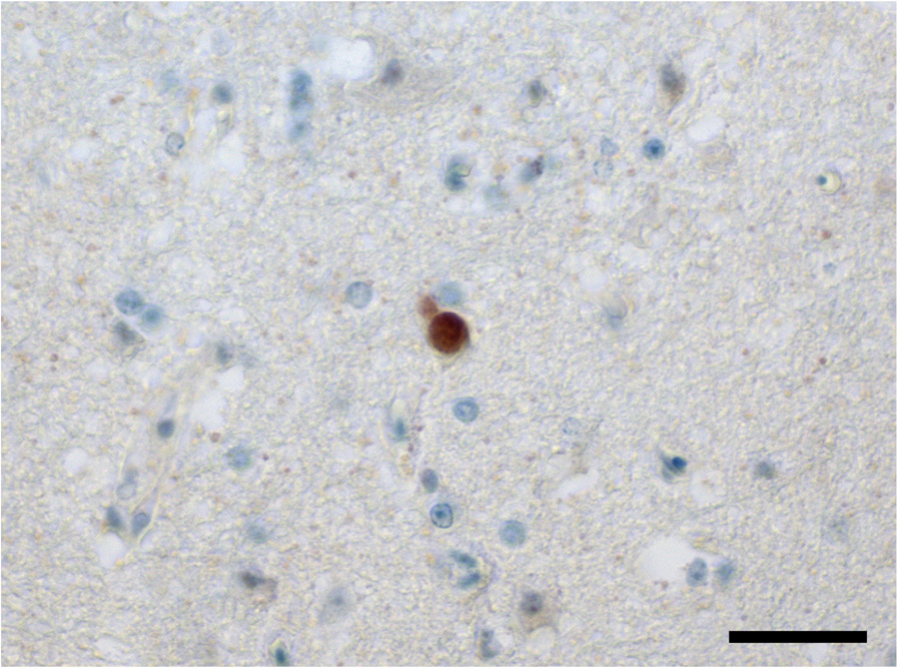
**Immunohistochemical labelling of a schizont with polyclonal antiserum to *S. calchasi *in the brain of pigeon no. 7 during the first phase of disease. **No associated cellular immune reaction or necrosis is discernible. In total, three schizonts were identified in consecutive section of the brain of this pigeon. Bar, 30 μm.

**Figure 3:**
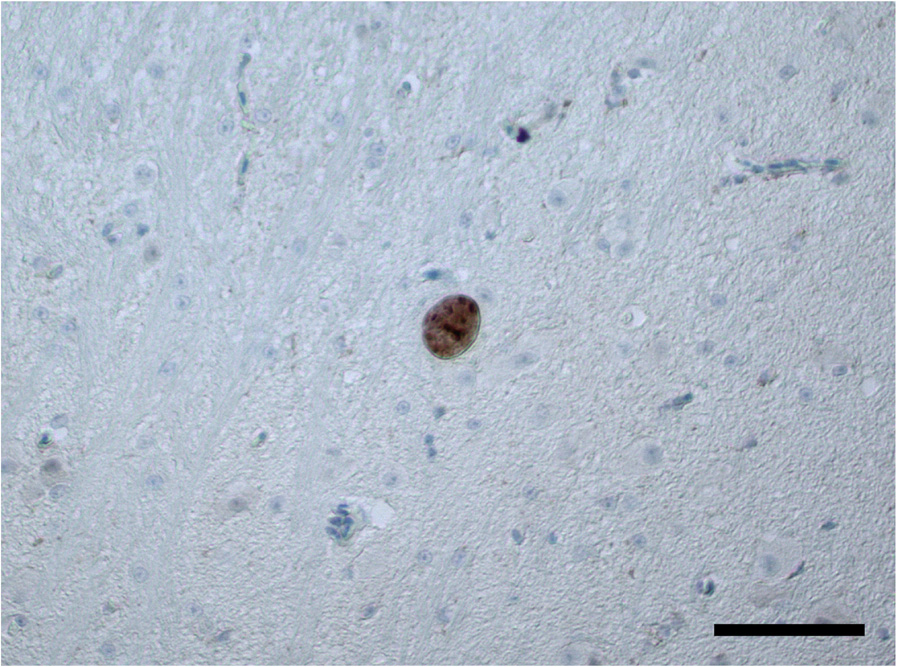
**Immunohistochemical labelling of a tissue cyst with polyclonal antiserum to *S. calchasi *in the brain of pigeon no. L60 during the second phase of disease. **No associated pathological lesions can be seen. In total, four tissue cysts were identified in the neuropil of this pigeon. Bar, 50 μm.

Immunohistochemical testing revealed that the anti-merozoite antibody is capable of labelling *S. calchasi* merozoites, schizonts and tissue cysts containing bradyzoites (data not shown). A positive labelling for *S. calchasi* was observed in the cerebrum of pigeons of group A and B. Four of eight pigeons of group A had rare schizonts in the neuropil (Figure 2 and Table 1). Mean schizont dimensions were 11.6 × 9.8 μm (*n* = 6, range = 7.3-13.4 × 6.5-13.2 μm). Three of seven pigeons of group B had few, randomly distributed tissue cysts located in areas of the neuropil not associated with pathological lesions or inflammatory cell reactions (Figure 3 and Table 1). The size of tissue cysts was in mean 19.0 × 15.1 μm (*n* = 14, range = 12.1-26.2 × 10.6-24.8 μm). All reference animals were negative for *S. calchasi* by immunohistochemistry.

**Figure 4:**
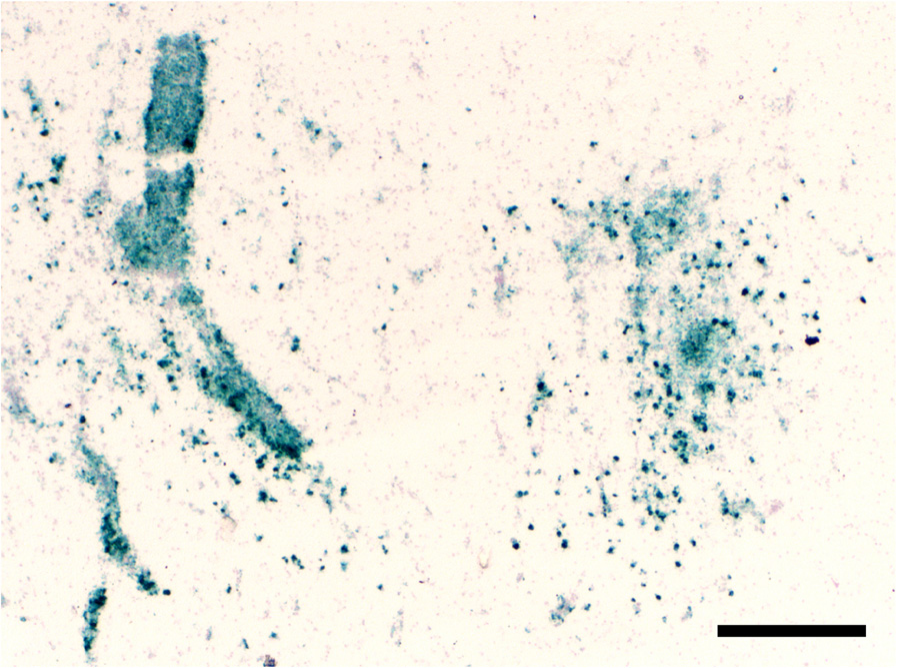
**Immunohistochemical detection of MHC-II of a pigeon with cerebral lesions in the second phase of disease. **Most prominent signaling is discernable in areas of perivasular cuffing of mononuclear cells (left) and areas of necrosis and gliosis (right). Bar, 300 μm.

**Figure 5:**
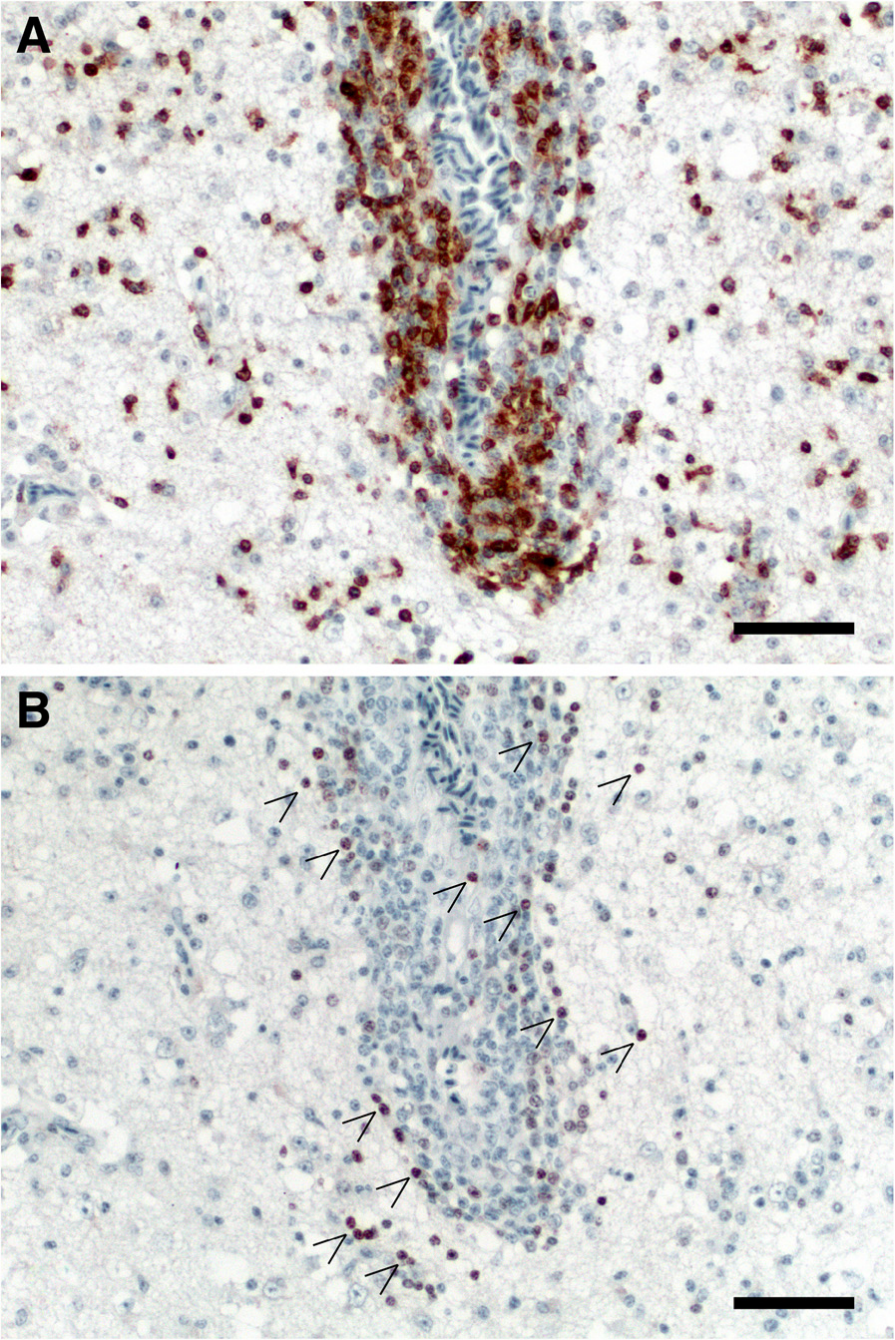
**Immunohistochemical demonstration of CD3+ T-cells (A), and Pax-5+ B cells (B) surrounding the same cerebral blood vessel of pigeon no. 12. **Note the dominance of T lymphocytes in perivascular cuffs and the neuropil. Arrows, positive stained B-cells. Bars 50 μm.

Areas with cerebral lesions including necrosis and gliosis as well as areas with mononuclear cell infiltration had strong MHC-II labelling in all pigeons of group B (Figure 4). Most mononuclear cells were strongly CD3 positive (T-lymphocytes) while only few cells were positive for Pax-5 (B-lymphocytes; Figure 5).

### Molecular detection of *S. calchasi*

**Figure 6:**
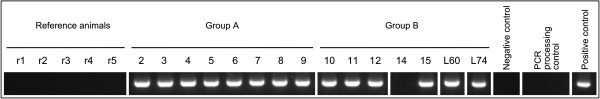
**PCR detection of *S. calchasi *DNA in brains of pigeons with specific nested primer pairs targeting the ITS1 region. **All brains of pigeons of group A (no. 2–9) and pigeons of group B (no. 10–12, 15, L60 and L74) except for no. 14 were positive. Five reference animals (r1-r5) used for RT-qPCR as well as negative controls and two processing controls were negative.

The nested PCR amplified DNA specific for *S. calchasi* in the brains of all pigeons of group A and all but one (pigeon no. 14) of group B (Figure 6). Reference animals were all negative. Processing controls and no template control also gave negative results.

### Cytokine expression profile

**Figure 7:**
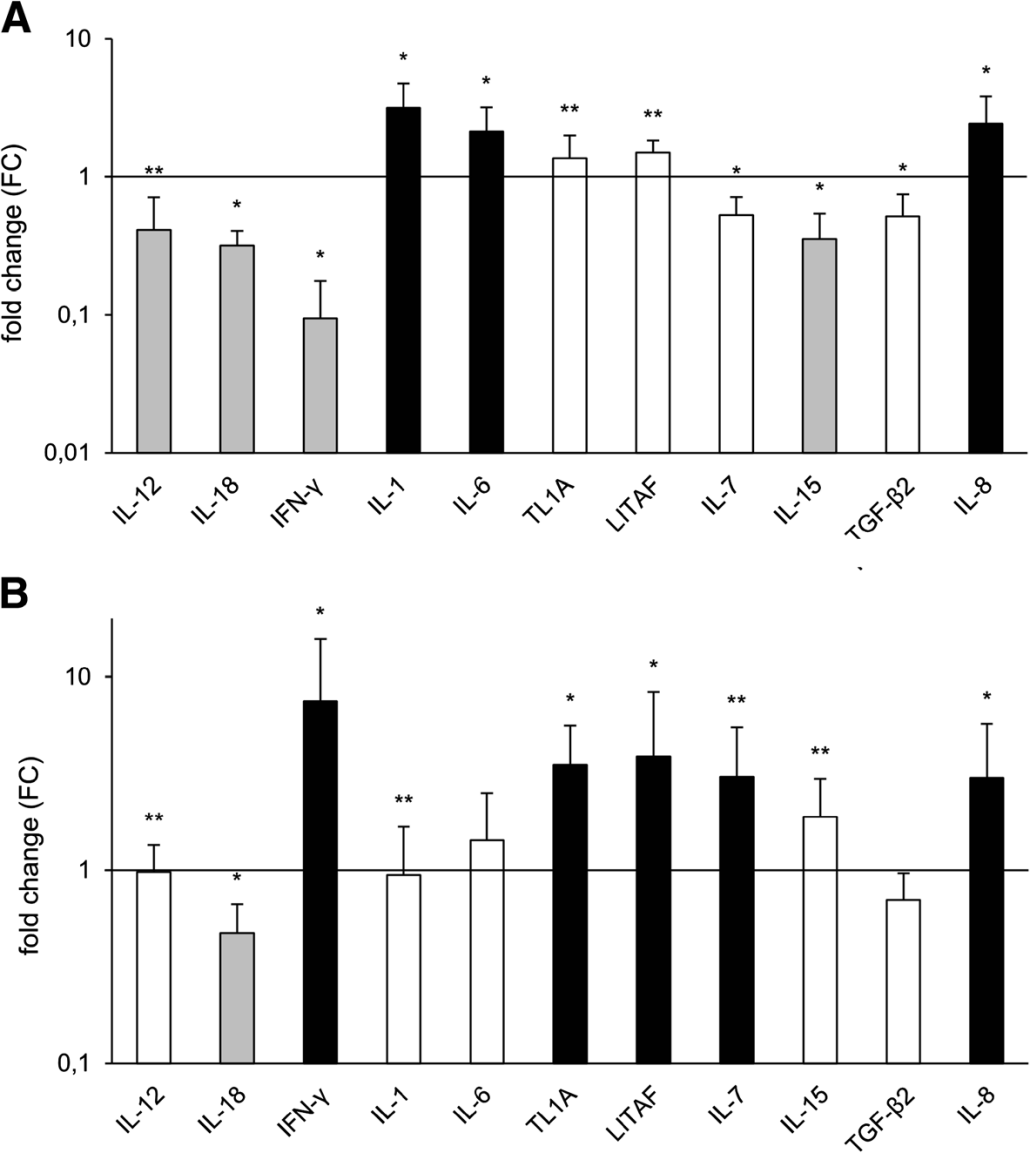
**Cytokine and chemokine expression profiles in the cerebrum from pigeons of group A (A) and group B (B) compared with non-infected pigeons (*n *= 5). **gray bar = down-modulated (FC < 0.5), white bar = unregulated (0.5 < FC <2), “black square” = up-modulated (FC ≥ 2). * = statistically significant at *p *≤ 0.05 by Mann–Whitney-U-test between group of infected and uninfected pigeons. ** = significant differences between group A and B.

Most stable reference genes for the cerebrum of pigeons of this study calculated by the GeNorm algorithm were RPL13, HMBS and beta-actin. For group A, mRNA expression levels of the Th-1 cytokines IL-12, IL-18 and IFN-γ were significantly down-modulated while the proinflammatory cytokines IL-1, IL-6, IL-15 and the chemokine IL-8 were significantly up-modulated when compared to the reference animals (Figure 7A). No regulation in mRNA expression levels was measured for TL1A, LITAF, IL-7 and TGF-β2. In contrast, for group B IFN-γ expression was significantly up-modulated while IL-18 was down-modulated and IL-12 was not regulated (Figure 7B). Furthermore, TL1A, LITAF and IL-7 were significantly up-modulated. IL-8 remained up-modulated, while IL-1 and IL-15 were unregulated when compared with the reference pigeons. IL-6 and TGF-β2 were also unregulated, but without statistical significance.

## Discussion

As demonstrated previously, *S. calchasi* is capable of causing a severe biphasic central nervous disease in the domestic pigeon [3]. Histologically, a severe lymphohistiocytic and necrotizing encephalitis was found in the late neurological phase of disease. So far, intralesional stages of the parasite had not been confirmed in the pigeon’s brains [5]. Because in tissue sections of cerebral sarcosporidiosis the parasitic load can be low or difficult to detect despite extensive pathological lesions [3,11], we generated and established an anti-merozoite antiserum against *S. calchasi*. The antibody reliably detected merozoites, schizonts and tissue cysts including bradyzoites by immunohistochemical analysis. Hereby we demonstrate that *S. calchasi* stages - although only very few - are present in about half of the brains in both clinical phases of PPE. The presence of *S. calchasi* DNA could be confirmed by PCR results for the ITS1 region from the cerebrum in all but one pigeon. Together the results indicate that *S. calchasi* is present in the brains of pigeons with PPE in both disease phases and may suggest a direct involvement of the parasite in the development of the cerebral lesions. However, since no direct association of parasitic stages with histopathological lesions was detected, an unknown immunopathological mechanism may trigger the extensive inflammatory lesions. This notion is underlined by one pigeon infected with 10^2^ sporocysts with a strong cellular immune response but negative results for parasite protein and DNA in the brain. To verify this, further experimental studies are needed since it cannot be ruled out that at the onset of central nervous signs all parasites are effectively eliminated by the immune system.

To further clarify the effect of *S. calchasi* on the cerebral immune response we established a novel panel of primers to measure the expression level of 11 key immune effector genes and 5 reference genes by RT-qPCR. We characterized an anti-parasite response profile of the host immune system. Most notably, during the schizogonic, first phase of disease the important Th1 cytokines IL-12, IL-18 and IFN-γ as well as IL-15 were significantly down-modulated. During this phase, schizonts were present in various organs, most prominently in the liver, spleen and endothelial cells [5]. In the brains, only very few schizonts were detected in the neuropil without discernible lesions or immune cell infiltrations, although the expression of the major pro-inflammatory cytokines IL-1 and IL-6 and the chemokine IL-8 were significantly up-modulated. This may suggest that similar to other members of the Apicomplexa, *S. calchasi* is capable of manipulating the IL-12/IL-18/IFN-γ axis to evade the cellular immune response [17].

Compared to closely related members of the Apicomplexa such as *Toxoplasma gondii* and *Neospora caninum* very little is known about the host immune response against *Sarcocystis* infection. IFN-γ has been shown to be essential for the protection against *S. neurona* neurological disease in mice [26]. IFN-γ is produced by T-cells, natural killer (NK) cells, monocytes and microglia in the brain [27]. While IFN-γ KO mice show severe neurological disease after experimental infection with *S. neurona*, SCID mice, which still have functional IFN-γ producing NK cells, only develop disease after treatment with neutralizing anti-IFN-γ antibodies [26]. Furthermore, it has been shown that CD8+ T-cells are critical for the protection from meningoencephalitis in C57BL/6 mice [28], while a humoral immune response seems to play no major role [29]. There is also first evidence that *S. neurona* may be capable of interfering with the cytokine signaling of the Th1 immune response. IFN-γ production was reduced in lymphocytes extracted from Equine protozoal meningoencephalitis (EPM) positive horses [16]. Isolated peripheral blood lymphocytes from horses with EPM that were co-cultured with SnSAG1 produced significantly less IFN-γ after 48 h [15] and the cell-mediated immune responses to SnSAG1 were significantly reduced in horses with EPM [16]. Together with the results of this study it is plausible to assume that *Sarcocystis* spp. in general may exhibit an immune evasion strategy that disrupts IFN-γ signaling. Since the Th1-biased immune response is of major importance in clearing infection with *T. gondii* and *N. caninum *[30,31], a balanced immune-modulation by the parasite is crucial for survival in its host. Impairment of the IL-12 and IFN-γ expression is therefore one central immune evasion strategy of *T. gondii*[17,18]. Furthermore, an impaired synthesis of IL-15 reduces the expression of IFN-γ which enhances the survival of *T. gondii*[32]. IL-15 also activates CD4+ and CD8+ T-cells, of which CD8+ T cells are stimulated to produce IFN-γ [33]. Interestingly, besides IL-12, IL-18 and IFN-γ, IL-15 was significantly down-modulated in the first phase of *S. calchasi* infection. This temporary immune suppression may, similar to *T. gondii*[30], facilitate *S. calchasi* to infect host cells and to replicate in the absence of a protective immune response.

In contrast to the first phase, the second neurological phase of disease is associated with a massive mononuclear cell infiltration and characterized by a markedly up-modulated IFN-γ expression. Notably, IL-12 and IL-18 as important inducers of IFN-γ were not up-modulated. IFN-γ is the key cytokine for the activation of mononuclear cells. This correlates well with the prominence of T-cells, the granulomatous character of the lesions and prominent MHC-II signaling in the pigeon brains. Chicken IFN-γ increases the expression of class II MHC on antigen presenting cells and directly activates macrophages and natural killer cells which can excrete further inflammatory effector molecules such as TNF-α that may destroy tissue. [34-36]. Since TNF-α has not been identified in the avian genome, we tested TL1A and LITAF which both were significantly up-modulated. Taken together this suggests an extensive Th1-biased T-cell driven immune response, which appears inappropriate in the light of a very low or absent parasite load.

Pigeons infected with *S. calchasi* depict neurological signs such as incoordination about eight weeks after infection when tissue cysts mainly located in skeletal muscles contain infectious bradyzoites [5]. This alteration in intermediate host behavior may lead to an increased predation rate by the final host, the Northern goshawk. In contrast to pigeons, several natural and aberrant hosts of avian *Sarcocystis* spp. (i.e. *S. neurona*) depict neurological signs associated with encephalitis only during the schizogonic phase which does not allow transmission to a final host [7,9,37,38]. Changes in intermediate host behavior as reported for common voles (*Microtus arvalis*) and lemmings (*Dicrostonyx richardsoni*) infected by birds of prey-transmitted *Sarcocystis cernae* and *Sarcocystis rauschorum*, respectively, have been suggested to enhance parasite transmission due to increased predation rates [39,40]. However, whether the change in behavior of pigeons induced by *S. calchasi* may be regarded as a parasite’s adaptation to enhance its fitness or simply as side effect due to a delayed-type hypersensitivity reaction requires further investigation to meet the manipulation hypothesis [41].

In conclusion, the observations of this study suggest that the Th-1 immune response during the schizogonic phase of the *S. calchasi* development is down-modulated in the intermediate host. The absence of a strong host’s cellular immune response in the pigeon may facilitate parasite evasion during acute disease and subsequent formation of tissue cysts. The results of this study further suggest that during the late central nervous phase of PPE a T-cell mediated delayed-type hypersensitivity reaction may cause the cerebral lesions.

## Competing interests

The authors declare that they have no competing interests.

## Authors’ contributions

Conceived and designed the experiments: PO, AM, ML, AG. Performed the experiments: PO, AM. Analyzed the data: PO, AM, RK, BK. Contributed reagents/ materials/ analysis tools: PO, ML, BK, AG. Wrote the paper: PO. All authors have read and approved the final manuscript.
